# The role of stress coping strategies for life impairments in ADHD

**DOI:** 10.1007/s00702-021-02311-5

**Published:** 2021-03-09

**Authors:** Steffen Barra, Andreas Grub, Michael Roesler, Petra Retz-Junginger, Florence Philipp, Wolfgang Retz

**Affiliations:** 1 Institute for Forensic Psychology and Psychiatry, Saarland University, Homburg, Germany; 2 Department of Developmental Psychology, Saarland University, Saarbruecken, Germany; 3 Department of Psychiatry and Psychotherapy, University Medical Center of the Johannes Gutenberg-University, Mainz, Germany

**Keywords:** Attention-deficit hyperactivity disorder, Burden, Distress, Regulation, Resources

## Abstract

Attention-deficit/hyperactivity disorder (ADHD) is commonly accompanied by functional impairments within personal, professional, and social context as well as further psychological distress. The role of perceived stress has been examined in this regard, although little is known about the influence of stress coping strategies. We examined the dynamics between ADHD symptomatology, stress coping strategies, and life impairments in a sample of 230 clinical and non-clinical adult individuals. ADHD was associated with low probability of choosing adaptive but high probability of implementing maladaptive stress coping strategies as well as with increased life impairments. Adaptive stress coping showed little effects beyond the associations between ADHD and life impairments, whereas some maladaptive coping strategies increased current life impairments over and above the effects of ADHD and further psychological distress. The present findings emphasize the need of professional support in adults with ADHD and the necessity to include stress coping strategies in respective treatment approaches aimed at reducing life impairments.

**Supplementary Information:**

The online version contains supplementary material available at 10.1007/s00702-021-02311-5.

Attention-deficit/hyperactivity disorder (ADHD) ranges among the most common psychiatric disorders in children and adolescents with world-wide prevalence estimations between 5 and 9% (Thomas et al. 2015; Cabral et al. 2020). Prospective studies indicate that ADHD often persists into adulthood with about 15% of childhood/youth cases showing full adult symptomatology and more than every second case showing at least some continuing ADHD symptoms (e.g., Faraone et al. 2006). Prevalence estimations for adult ADHD average around 2–3% (Fayyad et al. 2017). However, as adult ADHD is frequently accompanied by a variety of psychiatric/psychological comorbidity (Fayyad et al. 2017; Cabral et al. 2020), there is increased risk of not properly identifying ADHD as distinct psychopathology, thus, resulting in under-estimated prevalence rates (Bitter et al. 2019).

Besides co-existing psychiatric/psychological disorders, ADHD symptomatology also goes along with additional long-term functional impairments within the fields of education/workspace, family life, and further social context. For example, compared to individuals without ADHD, ADHD patients have been found to achieve lower academic/professional qualifications, to more frequently change jobs with higher rates of unemployment and sickness benefits, to have higher divorce rates and more frequent problems in social relations, to show increased risk for (persistent) crime involvement and mortality, and to report greater problems in daily life and lower overall quality of life (Barkley 2002; Barkley et al. 2004, 2006; Faraone and Biederman 2004; Danckaerts et al. 2010; Dalsgaard et al. 2015; Mohr-Jensen and Steinhausen 2016; Thorell et al. 2019; Arnold et al. 2020; Holst and Thorell 2020). Differences have been found between those diagnosed with ADHD by a clinician and those without diagnosis but relevant self-reported ADHD symptomatology (Pawaskar et al. 2020).

Perceived stress both in childhood and adulthood has been claimed to be an important factor associated with ADHD that contributes to elevated risk of comorbid disorders and further impairments (Daviss et al. 2009; Fuller-Thomson et al. 2014; Combs et al. 2015). Research points to a bi-directional relationship between stress and ADHD. Some researchers have stated that ADHD symptomatology may result from (early) stressful experiences due to changes in brain structure and functioning as well as cognitive adaptions in form of steady vigilance to potential threats (McCrory et al. 2012; Fuller-Thomson et al. 2014; Brown et al. 2017). On the other hand, ADHD appears to contribute to increased sensitivity for current stress, e.g., due to deviant stimulus processing. According to Barkley (1997), ADHD is based on reduced cognitive inhibition, which impedes maintaining attention to relevant cues while filtering irrelevant information. Potential over-stimulation may lead to impaired executive functioning in terms of cognitive, emotional, and behavioral control and, thus, promote increased rates of perceived stress. Previous studies have pointed to empirical evidence for reduced executive functioning related to ADHD symptomatology (Willcutt et al. 2005; Roberts et al. 2017). With respect to stress perception, studies on children, adolescents, and adults with ADHD found higher rates of subjectively perceived stress compared to healthy control participants in experimentally induced stress situations, whereas findings on objective, physiological markers (e.g., cortisol level) were inconclusive (Lackschewitz et al. 2008; Corominas-Roso et al. 2015; Isaksson et al. 2015; Raz and Leykin 2015). Moreover, increasing ADHD severity was linked to increasing rates of perceived stress (Combs et al. 2015; Miklósi et al. 2016).

Despite the empirical foundation concerning the relations between perceived stress, ADHD, and further life impairments, little is known about how to counteract unfavorable effects. More specifically, research on the use of stress coping strategies in adults with ADHD is scarce. The broader concept of emotion dysregulation is more frequently examined (Retz et al. 2012). With respect to emotion dysregulation in children, studies indicate that those diagnosed with ADHD rarely use adaptive coping strategies, whereas maladaptive coping strategies are implemented more frequently (Hampel and Desman 2006; Hampel et al. 2008; Schmitt et al. 2012). Concerning adults, Surman and colleagues (2013) have pointed to deficient emotional self-regulation in ADHD patients. Focusing more specifically on the concept of stress coping strategies, Young (2005) and Torrente et al. (2014) found that adults with ADHD were more prone to use maladaptive coping strategies than healthy controls, especially with respect to avoidance and escape. Whereas (Young 2005) stated that the choice of adaptive or maladaptive coping strategies may be dependent on the individual’s cognitive capability, (Torrente et al. 2014) found no associations between coping strategies and cognitive parameters. However, implications of both studies are impeded due to small sample sizes. Overbey et al. (2011) asked young adults about stress and respective coping strategies with regard to their intimate relationships. They found that higher self-reported ADHD symptomatology went along with increased rates of perceived stress and elevated risk of implementing maladaptive stress coping strategies.

In sum, existing research on the relations between ADHD, stress coping strategies, and life impairments is scarce and inconclusive. This is due to several factors, e.g., small sample sizes, focus on the broader concept of emotion dysregulation instead of more specifically on stress coping strategies, or the neglect of ADHD as relevant disorder in adulthood despite common comorbidity. However, to offer effective treatment approaches for individuals with ADHD that lower the effects of perceived stress and improve life impairments, it is of major importance to gain more sophisticated insights into the above-mentioned dynamics. Following the call of previous research (Torrente et al. 2014), the present study aimed at enlarging the current knowledge on the relations between ADHD, stress coping strategies, and life impairments facing the above-mentioned shortcomings of prior studies. We examined a large sample of adults for ADHD symptomatology/severity, a variety of adaptive and maladaptive stress coping strategies, and life impairments in different domains. To include common comorbidity, we also assessed further (comorbid) psychological distress. Based on the current literature, we expected associations of ADHD with elevated risks of (a) using maladaptive stress coping strategies and (b) life impairments. We further hypothesized that the use of adaptive stress coping strategies would buffer, and the use of maladaptive stress coping strategies would intensify the effects of ADHD on life impairments.

## Materials and methods

### Procedure and participants

The present study included data of 230 participants (females: *n* = 103, 44.8%; males: *n* = 127, 55.2%) with a mean age of 34.63 years (SD = 11.88; range = 17–65 years) and an average IQ of 107.88 (SD = 14.37; range = 83–145). Data were collected from a sample of adults from our ADHD outpatient consultation department for clarification of possible ADHD diagnosis (*n* = 136, 59.1%) as well as further participants (without suspicion of ADHD diagnosis) recruited on personal demand by the authors (*n* = 94, 40.9%). Study procedures were performed in accordance with the ethical standards of the Declaration of Helsinki. Participants were informed about study procedures and goals, voluntary participation, as well as anonymization of data. Written consent was provided.

### Questionnaires

ADHD self-rating questionnaire (ADHS-SB). Current ADHD symptomatology was assessed by the ADHD-SB, a self-rating questionnaire affiliated to the Homburger ADHD scales for adults (Rösler et al. 2008). The presence of a total of 22 items (18 symptom and four additional information items) is to be rated on a four-point Likert scale from 0 (= not present) to 3 (= severe). Item scores can be summed up to the three subscales Inattention (items 1–9), Hyperactivity (items 10–14), and Impulsivity (items 15–18). Moreover, a total current ADHD score can be calculated by adding up all 18 symptom items. Total current ADHD scores equal or above the cut-off value of 15 indicate clinically relevant current ADHD symptomatology.

Good psychometric properties were proven for the ADHD-SB, e.g., with test–retest reliabilities between *r* = 0.78 and 0.89 as well as internal consistencies between Cronbach’s *α* = 0.72 and 0.90 (Rösler et al. 2004). The total current ADHD score cut-off value of 15 showed a 77% sensitivity and a 75% specificity (Rösler et al. 2008).

Wender Utah Rating Scale-short version (WURS-k). Childhood ADHD was assessed by the WURS-k, another self-rating questionnaire affiliated to the Homburger ADHD scales for adults (Retz-Junginger et al. 2002; Rösler et al. 2008). For the retrospective estimation of ADHD symptoms within the age of 8–10 years, their presence is to be rated on 25 items (21 symptom items and four control items) using a five-point Likert scale from 0 (= not present) to 4 (= severe). The 21 symptom items are summed up to a total childhood ADHD score. Total childhood ADHD scores equal or above the cut-off value of 30 indicate clinically relevant childhood ADHD symptomatology.

Good psychometric properties were proven for the WURS-k, e.g., with a test–retest reliability of *r* = 0.90 (Rösler et al. 2008) and an internal consistency of Cronbach’s *α* = 0.91 (Stieglitz 2000). The total childhood ADHD score cut-off value of 30 showed a 85–93% sensitivity and a 76–92% specificity (Retz-Junginger et al. 2003, 2007).

Stress coping questionnaire (SVF). The German stress coping questionnaire (“Stressverarbeitungsfragebogen”, SVF; Janke et al. 1985) was used to describe cognitive and behavioral strategies aimed at maintaining or re-arranging psychological/psychosomatic stability after experiences of distress. Thereby, not only adaptive (short- and long-term stress-reducing) but also maladaptive (short-term stress-reducing but long-term stress-enhancing) strategies were assessed. The 114-item version of the SVF was used in the present study. Each participant was asked for his/her general tendency to react in terms of the given item when he/she feels impaired, negatively aroused, or imbalanced by someone scored on five-point Likert scale from 0 (= not at all) to 4 (= very likely). Items can be arranged to 19 subscales, representing (a) adaptive coping strategies (minimization, self-aggrandizement by comparison with others, denial of guild, distraction, substitute gratification, search for self-affirmation, situation control, reaction control, positive self-instructions, and need for social support), and (b) maladaptive coping strategies (avoidance, escape, social withdrawal, rumination, resignation, self-pity, self-blame, aggression, and drug use) (e.g., Ising et al. 2006). For the present study, we used *t* scores as provided by the manual, which were based on a German, non-clinical norm sample of 96 male and 104 female adults.

Good psychometric properties were proven for the SVF, e.g., with internal consistencies for most subscales of Cronbach’s *α* ≥ 0.79, except drug use with Cronbach’s *α* = 0.61 (Janke et al. 1985).

Sheehan disability scales. Participants rated the severity of current self-perceived impairments within the fields of work/school, social life, and family life/home responsibilities on the Sheehan Disability Scales (Sheehan 1983). Scores ranging from 0 to 10 can be clustered for each domain to represent no impairments (= 0), mild impairments (= 1–3), moderate impairments (= 4–6), severe impairments (= 7–9), and extreme impairments (= 10). Moreover, a total impairment score can be calculated by summing up ratings of all three scales.

Concerning psychometric properties, scales have been successfully implemented for reliable and valid assessment of functional impairments in ADHD patients (Coles et al. 2014; Pawaskar et al. 2020).

Symptom checklist 90-R (SCL-90-R). The German version of the SCL-90-R (Derogatis 1977; Franke and Derogatis 2002) was used to assess further (comorbid) psychological distress. Self-reports about the occurrence and severity of respective symptoms within the preceding 7 days are provided on 90 items that are scored on a five-point Likert scale ranging from 0 (= not at all) to 4 (= very strongly). Items can be assigned to nine subscales (somatization, obsessive compulsion, interpersonal sensitivity, depression, anxiety, hostility, phobic anxiety, paranoid ideation, and psychoticism). The global severity index (GSI) represents an average score across all items and, thus, serves as an indicator for overall self-perceived psychological distress. For the present study, we used *t* value transformations of subscale scores (Franke 1992) as well as the GSI.

Good psychometric properties were proven for the German SCL-90-R, e.g., with internal consistencies between Cronbach’s *α* = 0.79 and 0.98 (Franke et al. 1992).

Multiple-choice vocabulary intelligence test (MWT-B). To account for possible confounding due to general cognitive capability, participants took a short intelligence test in the form of the MWT-B (“Mehrfachwahl-Wortschatztest”; Lehrl 1977). This test measures verbal (crystallized) intelligence as an indicator of general cognitive capability, because it has been claimed not to be as susceptible to bias by psychological/psychiatric disturbances as fluid intelligence. Participants are given a sheet with a total of 37 lines, each of which containing five combinations of letters with only one displaying an actual word of the German language. This word must be identified. Based on the sum of correctly identified words, IQ values can be assigned according to the manual.

The MWT-B, which is commonly applied as German screening measure for cognitive capability, has been successfully implemented in ADHD research that has pointed to good psychometric properties, e.g., in terms of construct validity and test–retest reliability (*r* = 0.90; e.g., Conzelmann et al. 2010).

### Statistical analyses

Data were processed in IBM SPSS version 26.0. To investigate descriptive group differences between ADHD and non-ADHD participants, we conducted *χ*^2^-statistics for categorical variables (e.g., sex, clustered Sheehan Disability Scale ratings) and, for linear variables, *t* tests (e.g., age, IQ) as well as MANOVAs with post hoc Bonferroni or Games–Howell tests (SVF, SCL-90-R and linear Sheehan Disability Scale ratings) respecting potential interdependencies among variables. Partial eta-squared ($\eta_{{\text{p}}}^{2}$) was used as effect size to represent the percentage of variance in the dependent variable explained a the independent variable with values of 0.01, 0.06, and 0.14 representing small, medium, and large effects, respectively (Cohen 1988). For the examination of predictive effects of ADHD symptomatology on (1) the use of stress coping strategies, and (2) life impairments, as well as (3) potential buffering or intensifying effects of stress coping strategies on the associations between ADHD and life impairments irrespective of age, sex, and IQ, we performed multiple linear regression analyses (forced entry). First, we included the ADHD-SB scores as independent and the SVF subscale scores as dependent variables, with age, sex, and IQ as control variables (Table 4). Second (Tables 5, 6, online supplements), we used the ADHD-SB scores as independent and the linear Sheehan Disability Scale scores as dependent variable in a basic model (controlled for age, sex, and IQ), and then tested remaining predictive effects when further independent variables such as SVF subscale scores (Model a), SVF subscale and SCL-90-R GSI scores (Model b), and SVF subscale, SCL-90-R GSI scores, and the interaction term of ADHD-SB and SVF subscale scores were simultaneously considered. Accounting for *α*-error inflation due to multiple testing, we only considered findings with *p* values ≤ 0.001 as meaningful.

## Results

### Descriptives

Concerning ADHD, 46.5% of the total sample (*n* = 107) exceeded the WURS-k cut-off value of 30 points, thus indicating clinically relevant ADHD symptomatology in their childhoods. Of those, 105 (98.1%) participants had been referred from our ADHD outpatient consultation department. Moreover, 58.7% (*n* = 135) of all participants reported clinically relevant, current ADHD symptomatology (ADHD-SB score ≥ 15). Again, the majority of them (*n* = 119, 88.1%) came from our ADHD outpatient consultation department. For further analyses, we considered those participants to display a categorical ADHD index group who reported clinically relevant ADHD symptomatology both in childhood and at the current point in time (43.9%, *n* = 101; of those, *n* = 99, 98.0%, from our ADHD outpatient consultation department). ADHD and non-ADHD groups differed dimensionally on both ADHD-SB score (*M*_ADHD_ = 35.00, SD_ADHD_ = 8.22, *M*_non-ADHD_ = 11.92, SD_non-ADHD_ = 0.47, *F*(1, 228) = 377.54), *p* ≤ 0.001, *η*_p_^2^ = 0.62, and WURS-k score (*M*_ADHD_ = 46.33, SD_ADHD_ = 11.63, *M*_non-ADHD_ = 13.07, SD_non-ADHD_ = 11.09), *F*(1, 229) = 488.00,* p* ≤ 0.001, *η*_p_^2^ = 0.68. ADHD and non-ADHD subjects did not differ with respect to sex distribution, *χ*^2^ (1) = 0.04, *p* = 0.837, mean age, *t*(227.41) = 0.74, *p* = 0.461, or IQ, *t*(282) = 1.76, *p* = 0.08.

Table 1 presents differences between ADHD and non-ADHD participants on stress coping strategies. Compared to non-ADHD subjects, ADHD participants showed significantly lower scores on some but not all adaptive coping strategies; however, they had significantly higher scores on all maladaptive coping strategies.

**Table 1:** Mean differences of ADHD and non-ADHD participants on SVF stress coping strategies (t values)

		M (SD)				
SVF subscale		ADHD (*n* = 101)		Non-ADHD (*n* = 129)	*F* (1, 228)	$\eta_{{\text{p}}}^{2}$
Functional	Minimization	43.38 (10.09)		46.84 (8.43)	8.03**	0.03
	Self-aggrandizement by comparison with others	42.97 (10.82)		48.47 (9.48)	16.85***	0.07
	Denial of guild	48.11 (10.70)		48.30 (9.90)	0.02 n.s	0.00
	Distraction	48.13 (10.93)		48.69 (7.70)	0.21 n.s	0.00
	Substitute gratification	53.40 (10.40)		52.46 (9.23)	0.52 n.s	0.00
	Search for self-affirmation	48.69 (9.90)		49.33 (8.00)	0.29 n.s	0.00
	Situation control	40.96 (9.10)		46.74 (10.20)	19.96***	0.08
	Reaction control	42.62 (8.49)		44.32 (7.95)	2.42 n.s	0.01
	Positive self-instructions	37.59 (9.81)		44.31 (8.82)	29.77***	0.12
	Need for social support	52.56 (8.66)		52.82 (7.41)	0.06 n.s	0.00
Dysfunctional	Avoidance	53.53 (9.17)		48.72 (9.44)	15.10***	0.06
	Escape	62.04 (9.78)		48.95 (9.44)	82.56***	0.27
	Social withdrawal	58.76 (8.55)		46.72 (10.52)	87.18***	0.28
	Rumination	56.40 (9.28)		48.09 (10.56)	38.98***	0.15
	Resignation	62.10 (8.51)		48.75 (11.08)	100.20***	0.31
	Self-pity	57.03 (8.92)		47.70 (9.83)	55.34***	0.20
	Self-blame	58.94 (9.83)		48.28 (11.22)	56.95***	0.20
	Aggression	60.45 (8.49)		46.98 (9.62)	122.81***	0.35
	Drug use	54.83 (10.64)		45.27 (7.28)	65.22***	0.22

*n.s.* not significant, $\eta_{{\text{p}}}^{2}$ ≤ 0.01 = small, $\eta_{{\text{p}}}^{2}$ < 0.01 and ≤ .06 = medium, $\eta_{{\text{p}}}^{2}$ ≥ 0.14 = large. **p ≤ 0.01; ***p ≤ 0.001

Table 2 shows the differences between ADHD and non-ADHD groups on the Sheehan Disability Scales. ADHD participants not only displayed higher mean scores on all scales including the total impairment score, but they were also over-represented among those reporting severe and extreme impairments and under-represented among those reporting no or low impairments. Concerning further psychological distress measured by the SCL-90-R, ADHD participants showed higher scores than non-ADHD participants on all subscales as well as on the GSI (Table 3).

**Table 2:** Mean differences and distributions of ADHD and non-ADHD participants on the Sheehan disability scales

Sheehand disability scales		ADHD (*n* = 101)	Non-ADHD (*n* = 129)		*F*(1, 228)	$\eta_{{\text{p}}}^{2}$
M (SD)						
Work/school		7.07 (2.61)	3.31 (3.09)		95.96***	0.30
Social life		6.21 (2.52)	2.81 (2.84)		89.51***	0.28
Family life/home responsibilities		6.57 (2.48)	2.72 (2.70)		123.99***	0.35
Total impairment score		19.85 (5.46)	8.85 (7.82)		144.83***	0.39

					*χ*^2^(4)	
Impairment (*n*, *AR*)						
Work/school	No	3 (− 5.1)	37 (5.1)		63.45***	
	Low	10 (− 3.9)	28 (3.9)			
	Moderate	18 (0.0)	23 (0.0)			
	Severe	49 (4.8)	24 (− 4.8)			
	Extreme	21 (4.0)	5 (− 4.0)			
Social life	No	1 (− 6.3)	44 (6.3)		63.08***	
	Low	18 (− 2.0)	38 (2.0)			
	Moderate	27 (1.0)	27 (− 1.0)			
	Severe	47 (5.3)	19 (− 5.3)			
	Extreme	8 (2.8)	1 (− 2.8)			
Family life/home responsibilities	No	1 (− 6.1)	42 (6.1)		78.05***	
	Low	13 (− 3.7)	44 (3.7)			
	Moderate	30 (1.7)	26 (− 1.7)			
	Severe	47 (5.6)	17 (− 5.6)			
	Extreme	10 (3.7)	0 (− 3.7)			

*AR*   adjusted residual, AR ≤ − 2.0 or ≥ 2.0 represent significant deviations from expected cell frequencies

$\eta_{{\text{p}}}^{2}$ ≤ .01 = small, $\eta_{{\text{p}}}^{2}$< .01 and ≤ .06 = medium, $\eta_{{\text{p}}}^{2}$ ≥ .14 = large. ***p ≤ 0.001

**Table 3:** Mean differences of ADHD and non-ADHD participants on the SCL-90-R (*T*-values)

	*M* (*SD*)						*n* (%) with *T* ≥ 60		
SCL-90-R subscale	ADHD (*n* = 101)		Non-ADHD (*n* = 129)	*F* (1, 225)	$\eta_{{\text{p}}}^{2}$		ADHD (*n* = 101)	Non-ADHD (*n* = 129)	*χ*^2^(1)
Somatization	61.49 (13.68)		53.06 (12.64)	23.17***	0.09		56 (55.4)	48 (37.2)	7.61**
Obsessive compulsion	71.36 (9.33)		50.43 (13.77)	170.43***	0.43		89 (88.1)	42 (32.6)	71.33***
Interpersonal sensitivity	65.09 (13.44)		48.47 (13.02)	88.77***	0.28		66 (65.3)	27 (20.9)	46.40***
Depression	68.99 (12.14)		51.17 (14.80)	95.08***	0.30		82 (81.2)	40 (31.0)	57.27***
Anxiety	66.53 (12.47)		49.29 (12.28)	109.08***	0.33		75 (74.3)	28 (21.7)	63.27***
Hostility	65.40 (12.26)		49.41 (12.37)	94.38***	0.30		73 (72.3)	28 (21.7)	58.82***
Phobic anxiety	62.44 (14.03)		48.91 (9.44)	74.79***	0.25		60 (59.4)	22 (17.1)	44.29***
Paranoid ideation	63.52 (12.96)		49.87 (11.32)	71.72***	0.24		66 (65.3)	30 (23.3)	41.27***
Psychoticism	62.98 (12.93)		49.77 (10.85)	70.03***	0.24		61 (60.4)	31 (24.0)	31.21***
GSI	67.97 (14.85)		50.19 (15.17)	78.43***	0.26		79 (78.2)	36 (27.9)	57.35***

$\eta_{{\text{p}}}^{2}$ ≤ .01 = small, $\eta_{{\text{p}}}^{2}$ < .01 and ≤ .06 = medium, $\eta_{{\text{p}}}^{2}$ ≥ .14 = large. **p ≤ 0.01, ***p ≤ 0.001

### Associations between ADHD symptomatology, stress coping strategies, and life impairments

Considering the above-mentioned differences between ADHD and non-ADHD participants on stress coping strategies, we further found negative predictive associations of current ADHD severity with adaptive stress coping strategies as well as positive predictive associations of current ADHD severity with maladaptive stress coping strategies (controlled for age, sex, and IQ; Table 4).

**Table 4:** Predictive associations between ADHD and stress coping strategies

Predictor	Outcome	*B*	95%*CI*	*β*	*p*
ADHD-SB score	Minimization	− 0.17	− 0.25, − 0.08	− 0.26	≤ 0.001
	Self-aggrandizement by comparison with others	− 0.27	− 0.36, − 0.18	− 0.38	≤ 0.001
	Situation control	− 0.24	− 0.33, − 0.15	− 0.35	≤ 0.001
	Positive self-instructions	− 0.27	− 0.36, − 0.19	− 0.40	≤0.001
	Avoidance	0.22	0.13, 0.31	0.33	≤ 0.001
	Escape	0.53	0.43, 0.62	0.61	≤ 0.001
	Social withdrawal	0.49	0.41, 0.57	0.63	≤ 0.001
	Rumination	0.35	0.26, 0.44	0.48	≤ 0.001
	Resignation	0.54	0.46, 0.63	0.66	≤ 0.001
	Self-pity	0.37	0.29, 0.46	0.51	≤ 0.001
	Self-blame	0.44	0.35, 0.53	0.54	≤ 0.001
	Aggression	0.50	0.41, 0.58	0.64	≤ 0.001
	Drug use	0.36	0.28, 0.44	0.53	≤ 0.001

All analyses controlled for age, sex, and IQ

Under the control of potential effects of age, sex, and IQ, current ADHD severity proved to be a significant predictor for life impairments even under consideration of stress coping strategies (Tables 5, 6, Model a) as well as further (comorbid) psychological distress (Tables 5, 6, Model b); however, no meaningful interactions between ADHD severity and stress coping strategies were found (Tables 5, 6, Model c). Adaptive stress coping strategies did not show any meaningful predictive effects on overall life impairments over and above the effects of current ADHD severity and further (comorbid) psychological distress (Table 5, Models a, b), although some tendencies emerged for negative associations, e.g., regarding positive self-instructions. Among maladaptive stress coping strategies (Table 6), escape, social withdrawal, and resignation were positively associated with overall life impairments over and above the effects of ADHD severity and further (comorbid) psychological distress (Table 6, Models a, b). Tendencies for similar effects of escape and resignation on work/school impairments emerged (Online Supplement 1). Focusing on social life impairments (Online Supplement 2), minimization and reaction control contained meaningful negative predictive effects over and above those of ADHD and psychological distress and a respective tendency was found for positive self-instructions. Concerning maladaptive strategies, social withdrawal proved to be positively related to increased social impairment beyond ADHD severity and further (comorbid) psychological distress, whereas a tendency emerged for escape. Social withdrawal was also positively related to family impairments (Online Supplement 3) by tendency. No significant interaction effects including current ADHD symptomatology and stress coping strategies on life impairments emerged.

**Table 5:** Associations between ADHD, adaptive stress coping strategies, and total life impairments

	Δ*R*^*2*^	*p*	Predictor	*B*	95% CI	*β*	*p*
	0.56	≤ .001	ADHD	0.45	0.40, 0.51	0.75	≤ 0.001
Model a	0.01	.008	ADHD	0.43	0.38, 0.49	0.72	≤ 0.001
			Minimization	− 0.12	− 0.20, − 0.03	− 0.12	0.008
Model b	0.06	≤ .001	ADHD	0.31	0.25, 0.38	0.52	≤ 0.001
			Minimization	− 0.11	− 0.18, -0.03	− 0.11	0.010
			SCL-90-R GSI	0.17	0.12, 0.22	0.34	≤ 0.001
Model c	0.00	.288	ADHD*minimization	0.00	0.00, 0.01	0.23	0.288
Model a	0.01	.042	ADHD	0.43	0.37, 0.49	0.72	≤ 0.001
			self-aggrandizement by comparison with others	− 0.09	− 0.1, 0.00	− 0.10	0.042
Model b	0.06	≤ .001	ADHD	0.32	0.25, 0.38	0.53	≤ 0.001
			self-aggrandizement by comparison with others	− 0.04	− 0.12, 0.03	− 0.05	0.262
			SCL-90-R GSI	0.17	0.11, 0.22	0.33	≤ 0.001
Model c	0.00	.619	ADHD*self-aggrandizement by comparison with others	0.00	− 0.01, 0.00	− 0.10	0.619
Model a	0.00	.323	ADHD	0.45	0.40, 0.51	0.75	≤ 0.001
			denial of guild	− 0.04	− 0.12, 0.04	− 0.05	0.323
Model b	0.07	≤ .001	ADHD	0.32	0.26, 0.39	0.54	≤ 0.001
			denial of guild	− 0.03	− 0.11, 0.04	− 0.04	0.356
			SCL-90-R GSI	0.17	0.12, 0.23	0.34	≤ 0.001
Model c	0.00	.449	ADHD*denial of guild	0.00	0.00, 0.01	0.15	0.449
Model a	0.01	.102	ADHD	0.45	.40, .51	0.75	≤ 0.001
			distraction	− 0.07	− 0.16, 0.01	− .08	0.102
Model b	0.07	≤ .001	ADHD	0.32	0.26, 0.39	0.54	≤ 0.001
			distraction	− 0.09	− 0.17, − 0.01	− .09	0.035
			SCL-90-R GSI	0.18	0.12, 0.23	0.35	≤ 0.001
Model c	0.00	.531	ADHD*distraction	0.00	− 0.01, 0.00	− 0.13	0.531
Model a	0.00	.430	ADHD	0.45	0.40, 0.51	0.76	≤ 0.001
			substitute gratification	0.03	− 0.05, 0.12	0.04	0.430
Model b	0.07	≤ .001	ADHD	0.33	0.26, 0.39	0.54	≤ 0.001
			substitute gratification	0.01	− 0.07, 0.09	0.01	0.807
			SCL-90-R GSI	0.17	0.12, 0.23	0.34	≤ 0.001
Model c	0.01	.026	ADHD*substitute gratification	0.01	0.00, 0.01	0.53	0.026
Model a	0.00	.282	ADHD	0.45	0.39, 0.50	0.75	≤ 0.001
			search for self-affirmation	− 0.05	− 0.14, 0.04	− .05	0.282
Model b	0.07	≤ .001	ADHD	0.32	0.25, 0.38	0.53	≤ 0.001
			search for self-affirmation	− 0.07	− 0.15, 0.01	− 0.07	0.103
			SCL-90-R GSI	0.18	0.12, 0.23	0.35	≤ 0.001
Model c	0.00	.295	ADHD*search for self-affirmation	0.00	0.00, 0.01	0.27	0.295
Model a	0.01	.010	ADHD	0.42	0.37, 0.48	0.71	≤ 0.001
			situation control	− 0.11	− 0.20, -0.03	− 0.13	0.010
Model b	0.06	≤ .001	ADHD	0.31	0.24, 0.37	0.51	≤ 0.001
			situation control	− 0.09	− 0.17, − 0.01	− 0.10	0.029
			SCL-90-R GSI	0.17	0.11, 0.22	0.33	≤ 0.001
Model c	0.01	.066	ADHD*situation control	0.01	0.00, 0.01	0.36	0.066
Model a	0.01	.013	ADHD	0.44	0.39, 0.50	0.74	≤ 0.001
			reaction control	− 0.12	− 0.22, -0.03	− 0.12	0.013
Model b	0.07	≤ .001	ADHD	0.32	0.25, 0.38	0.53	≤ 0.001
			reaction control	− 0.12	− 0.20, − 0.03	− 0.11	0.011
			SCL-90-R GSI	0.17	0.12, 0.23	0.34	≤ 0.001
Model c	0.00	.322	ADHD* reaction control	0.00	0.00, 0.01	0.22	0.322
Model a	0.03	≤ .001	ADHD	0.41	0.35, 0.47	0.68	≤ 0.001
			positive self-instructions	− 0.16	− 0.24, − 0.08	− 0.18	≤ 0.001
Model b	0.06	≤ .001	ADHD	0.30	0.24, 0.37	0.50	≤ 0.001
			positive self-instructions	− 0.12	− 0.20, − 0.04	− 0.14	0.002
			SCL-90-R GSI	0.16	0.11, 0.21	0.32	≤ 0.001
Model c	0.00	.841	ADHD* positive self-instructions	0.00	− 0.01, 0.01	0.04	0.841
Model a	0.00	.671	ADHD	0.45	0.40, 0.51	0.75	≤ 0.001
			need for social support	− 0.02	− 0.13, 0.08	− 0.02	0.671
Model b	0.07	≤ 0.001	ADHD	0.32	0.26, 0.39	0.54	≤ 0.001
			need for social support	− 0.03	− 0.13, 0.06	− 0.03	0.484
			SCL-90-R GSI	0.17	0.12, 0.23	0.34	≤ 0.001
Model c	0.00	0.825	ADHD* need for social support	0.00	− 0.01, 0.01	0.07	0.825

All models include age, sex, and IQ as control variables. Outcome was the Sheehan Disability Scale total score. Model c includes all mentioned variables; only the interaction term is displayed

**Table 6:** Associations between ADHD, maladaptive stress coping strategies, and total life impairments

	Δ*R*^2^	*p*	Predictor	*B*	95%CI	*β*	*p*
	.56	≤ .001	ADHD	0.45	0.40, 0.51	0.75	≤ 0.001
Model a	.01	.023	ADHD	0.43	0.37, 0.49	0.72	≤ .001
			Avoidance	0.10	0.01, 0.19	0.11	0.023
Model b	.06	≤ .001	ADHD	0.32	0.23, 0.38	0.53	≤ 0.001
			Avoidance	0.05	− 0.03, 0.13	0.06	0.219
			SCL-90-R GSI	0.17	0.11, 0.22	0.33	≤ 0.001
Model c	.00	.116	ADHD*avoidance	0.00	0.00, 0.01	0.38	0.116
Model a	.05	≤ .001	ADHD	0.35	0.28, 0.42	0.59	≤ 0.001
			Escape	0.19	0.12, 0.26	0.27	≤ 0.001
Model b	.04	≤ .001	ADHD	0.28	0.21, 0.34	0.46	≤ 0.001
			Escape	0.13	0.06, 0.21	0.20	≤ 0.001
			SCL-90-R GSI	0.14	0.09, 0.20	0.28	≤ 0.001
Model c	.00	.681	ADHD*escape	0.00	0.00, 0.01	0.10	0.681
Model a	.05	≤ .001	ADHD	0.35	0.28, 0.41	0.58	≤ 0.001
			Social withdrawal	0.21	0.13, 0.30	0.28	≤ 0.001
Model b	.05	≤ .001	ADHD	0.27	0.20, 0.34	0.44	≤ 0.001
			Social withdrawal	0.16	0.08, 0.24	0.21	≤ 0.001
			SCL-90-R GSI	0.15	0.09, 0.20	0.29	≤ 0.001
Model c	.00	.980	ADHD*social withdrawal	0.00	− 0.01, 0.01	− 0.01	0.980
Model a	.01	.037	ADHD	0.42	0.36, 0.48	0.70	≤ 0.001
			Rumination	0.09	0.01, 0.17	0.11	0.037
Model b	.06	≤ .001	ADHD	0.32	0.25, 0.39	0.53	≤ 0.001
			Rumination	0.03	− 0.06, 0.10	0.03	0.543
			SCL-90-R GSI	0.17	0.11, 0.23	0.33	≤ 0.001
Model c	.00	.251	ADHD*rumination	0.00	0.00, 0.01	0.31	0.251
Model a	.05	≤ .001	ADHD	0.34	0.27, 0.41	0.57	≤ 0.001
			Resignation	0.21	0.12, 0.29	0.28	≤ 0.001
Model b	.04	≤ .001	ADHD	0.27	0.20, 0.34	0.46	≤ 0.001
			Resignation	0.14	0.06, 0.22	0.19	≤ 0.001
			SCL-90-R GSI	0.14	0.08, 0.20	0.28	≤ 0.001
Model c	.00	.579	ADHD*resignation	0.00	0.00, 0.01	0.16	0.579
Model a	.02	.002	ADHD	0.40	0.34, 0.47	0.67	≤ 0.001
			Self-pity	0.13	0.05, 0.22	0.16	0.002
Model b	.05	≤ .001	ADHD	0.32	0.25, 0.38	0.53	≤ 0.001
			Self-pity	0.04	− 0.04, 0.13	0.05	0.325
			SCL-90-R GSI	0.16	0.10, 0.22	0.32	≤ 0.001
Model c	.00	.853	ADHD*self-pity	0.00	− 0.01, 0.01	0.05	0.853
Model a	.01	.035	ADHD	0.41	0.35, 0.48	0.69	≤ 0.001
			Self-blame	0.09	0.01, 0.17	0.12	0.035
Model b	.06	≤ .001	ADHD	0.32	0.25, 0.39	0.53	≤ 0.001
			Self-blame	0.02	− 0.06, 0.10	0.02	0.648
			SCL-90-R GSI	0.17	0.11, 0.23	0.33	≤ 0.001
Model c	.00	.756	ADHD*self-blame	0.00	0.00, 0.01	0.09	0.756
Model a	.02	.002	ADHD	0.38	0.31, 0.45	0.64	≤ 0.001
			Aggression	0.14	0.05, 0.23	0.18	0.002
Model b	.06	≤ .001	ADHD	0.29	0.21, 0.36	0.48	≤ 0.001
			Aggression	0.10	0.01, 0.18	0.12	0.025
			SCL-90-R GSI	0.16	0.11, 0.22	0.32	≤ 0.001
Model c	.00	.696	ADHD*aggression	0.00	− 0.01, 0.00	− 0.11	0.696
Model a	.00	.432	ADHD	0.44	0.37, 0.50	0.73	≤ 0.001
			drug use	0.04	− 0.06, 0.13	0.04	0.432
Model b	.07	≤ .001	ADHD	0.33	0.26, 0.40	0.55	≤ 0.001
			Drug use	− 0.02	− 0.11, 0.07	− 0.02	0.665
			SCL-90-R GSI	0.18	0.12, 0.23	0.35	≤ 0.001
Model c	.00	.709	ADHD*drug use	0.00	− 0.01, 0.01	− 0.11	0.709

All models include age, sex, and IQ as control variables. Outcome was the Sheehan Disability Scale total score. Model c includes all mentioned variables; only the interaction term is displayed

## Discussion

The present study contributes to previous research by shedding light into the dynamics between ADHD, stress coping strategies, and life impairments. The examination of these factors in a large sample of adults led to the following results that allow the deduction of important implications for treatment approaches aimed at reducing life impairments in adults with ADHD.

Consistent with our expectations, ADHD participants used some adaptive stress coping strategies less often but implemented all maladaptive stress coping strategies particularly more often than non-ADHD individuals. Furthermore, irrespective of age, sex, or cognitive capability, increased ADHD severity lowered the probability of engaging in adaptive stress coping strategies but enhanced the risk of choosing maladaptive stress coping strategies. These findings support and extent results from previous studies that emphasized the tendency of adults with ADHD to get involved in maladaptive stress coping (Young 2005; Overbey et al. 2011; Torrente et al. 2014). Adaptive stress coping, however, appears of specific importance in ADHD considering the relations of perceived stress and symptomatology (Combs et al. 2015; Miklósi et al. 2016). Failure to respond adequately to stress may put individuals with ADHD at risk of being captured in a vicious cycle in which stress perception and ADHD symptomatology trigger and reinforce each other.

Also as hypothesized, ADHD participants showed increased life impairments on all Sheehan Disability Scales as well as elevated further (comorbid) psychological distress (SCL-90-R) compared to non-ADHD subjects. ADHD severity proved to be a consistent predictor of increased impairments over and above the effects of age, sex, IQ, further (comorbid) psychological distress, and stress coping strategies. These results are in line with those of previous research (Pitts et al. 2015; Holst and Thorell 2020) and highlight the far-reaching personal and social issues affiliated with ADHD as well as the neediness of individuals with ADHD to receive adequate support.

The role of stress coping strategies for the associations between ADHD and life impairments appears more complex. Most strategies did not exert any meaningful effects on life impairments beyond those of ADHD and further (comorbid) psychological distress. However, independent of ADHD severity, the implementation of some maladaptive strategies, e.g., escape, resignation, and social withdrawal, appeared to increase life impairments, whereas some adaptive strategies, e.g., minimization, reaction control, and positive self-instructions, tended toward the reduction of life impairments, especially related to social contexts. These findings emphasize the need to counteract maladaptive stress coping and foster the implementation of adaptive stress coping strategies to reduce life impairments. Yet, as we did not find any interactions in terms of buffering or intensifying impacts of stress coping strategies on the associations between ADHD severity and life impairments (contrary to our expectations), the potential interdependency of ADHD and stress coping strategies for reducing or increasing life impairments remains a subject for further research. Still, it is imperative to include and promote the acquirement of adaptive stress coping strategies in the treatment of adults with ADHD to break through the above-mentioned vicious cycle of perceived stress and ADHD, and offer functional means to reduce the risk of further life impairments.

### Strengths and limitations

Several strengths and some limitations must be considered when interpreting the current findings. As demanded by previous research (Torrente et al. 2014), we examined the dynamics between ADHD, stress coping strategies, and life impairments in a large sample of adults. Data were based on self-reports of childhood and current ADHD symptoms, which allowed for the consideration of ADHD symptomatology/severity meeting claims of previous research not only to rely on clinical diagnoses but also include dimensional (subclinical) representations of ADHD (Philipp-Wiegmann et al. 2018). Although we aimed at examining a sample that offered a broader range of dimensional ADHD symptomatology/severity by including both clinically and non-clinically referred participants, our sample was not representative of the general population and some selection bias (e.g., due to self-initiated introduction to our ADHD outpatient consultation department) cannot be excluded. Moreover, the sole reliance on self-reported data holds the risk of subjective bias, e.g., in terms of under/over-reporting of symptomatology or feigning. This is important to consider, especially because assignments to ADHD and non-ADHD subsamples were based on self-reported symptomatology only. Also, accounting for clinician-administered ADHD diagnoses appeared to be important in the investigation of life impairments associated with ADHD, as differences were found in the degrees of impairment between clinically diagnosed adults and adults with clinically relevant, self-reported ADHD symptomatology (Pawaskar et al. 2020). However, we were not able to include clinician-administered diagnoses in the present study. Regarding our research design, findings are based on cross-sectional, correlational data, which prevent any causal inferences. Despite the consideration of a variety of adaptive and maladaptive stress coping strategies that allowed for sophisticated insights into their associations with ADHD and life impairments, future studies could been strengthened by the (longitudinal) inclusion of subjective and objective measures of psychological/psychiatric distress and perceived stress, which was, however, beyond the scope of the present study.

## Conclusion and implications

In conclusion, the current findings provide further evidence that ADHD is associated with increased life impairments in multiple domains. Adaptive stress coping strategies were seldomly applied by adults with ADHD and showed little effects on these associations, whereas maladaptive stress coping strategies were frequent and some of them (e.g., social withdrawal) appeared to increase current life impairments. With respect to the potential long-term risks related to maladaptive coping, the acquirement and adequate application of adaptive stress coping strategies is of major importance for individuals with ADHD. Thus, studies and, especially, practitioners working with individuals with ADHD must not neglect to assess their clients’ perceived stress and the coping strategies already in use, but need to provide them with respective treatment approaches that support them in the successful implementation of stress-reducing techniques.

## Supplementary Information

Below is the link to the electronic supplementary material.

Supplementary file1 (DOCX 26 KB)

Supplementary file2 (DOCX 26 KB)

Supplementary file3 (DOCX 26 KB)
