# Supplementary material for: The role of stress coping strategies for life impairments in ADHD
Source: J Neural Transm (Vienna). 2021 Mar 9;128(7):981–92. doi: 10.1007/s00702-021-02311-5 (PMC8295144; doi:10.1007/s00702-021-02311-5)
Supplement: Supplementary file 1 — Supplementary file1 (DOCX 26 KB) [file 702_2021_2311_MOESM1_ESM.docx]

Online Supplement 3: Associations between ADHD, stress coping strategies, and Sheehan Disability Scale: impairments in family life/home responsibilities.

|  | Δ*R²* | *p* | Predictor | *B* | 95%*CI* | ß | *p* |
| --- | --- | --- | --- | --- | --- | --- | --- |
|  | .50 | ≤ .001 | ADHD | 0.16 | 0.13, 0.18 | .71 | ≤ .001 |
| Model a | .01 | .100 | ADHD | 0.15 | 0.13, 0.17 | .69 | ≤ .001 |
|  |  |  | minimization | -0.03 | -0.06, 0.01 | -.08 | .100 |
| Model b | .06 | ≤ .001 | ADHD | 0.11 | 0.08, 0.13 | .49 | ≤ .001 |
|  |  |  | minimization | -0.02 | -0.06, 0.01 | -.07 | .137 |
|  |  |  | SCL-90-R GSI | 0.06 | 0.04, 0.08 | .32 | ≤ .001 |
| Model c | .01 | .076 | ADHD*minimization | 0.00 | 0.00, 0.00 | .42 | .076 |
| Model a | .00 | .512 | ADHD | 0.15 | 0.13, 0.18 | .69 | ≤ .001 |
|  |  |  | self-aggrandizement by comparison with  others | -0.01 | -0.04, 0.02 | -.04 | .512 |
| Model b | .06 | ≤ .001 | ADHD | 0.11 | 0.09, 0.14 | .51 | ≤ .001 |
|  |  |  | self-aggrandizement by comparison with  others | 0.00 | -0.03, 0.04 | .01 | .801 |
|  |  |  | SCL-90-R GSI | 0.06 | 0.04, 0.08 | .33 | ≤ .001 |
| Model c | .00 | .567 | ADHD* self-aggrandizement by comparison  with others | 0.00 | 0.00, 0.00 | .12 | .567 |
| Model a | .00 | .522 | ADHD | 0.16 | 0.13, 0.18 | .71 | ≤ .001 |
|  |  |  | denial of guild | -0.01 | -0.04, 0.02 | -.03 | .522 |
| Model b | .06 | ≤ .001 | ADHD | 0.11 | 0.09, 0.14 | .51 | ≤ .001 |
|  |  |  | denial of guild | -0.01 | -0.04, 0.02 | -.03 | .580 |
|  |  |  | SCL-90-R GSI | 0.06 | 0.04, 0.08 | .32 | ≤ .001 |
| Model c | .01 | .124 | ADHD*denial of guild | 0.00 | 0.00, 0.00 | .32 | .124 |
| Model a | .00 | .849 | ADHD | 0.16 | 0.13, 0.18 | .71 | ≤ .001 |
|  |  |  | distraction | 0.00 | -0.03, 0.04 | .01 | .849 |
| Model b | .06 | ≤ .001 | ADHD | 0.11 | 0.09, 0.14 | .51 | ≤ .001 |
|  |  |  | distraction | 0.00 | -0.03, 0.03 | .00 | .935 |
|  |  |  | SCL-90-R GSI | 0.06 | 0.04, 0.08 | .32 | ≤ .001 |
| Model c | .00 | .808 | ADHD* distraction | 0.00 | 0.00, 0.00 | -.06 | .808 |
| Model a | .00 | .381 | ADHD | 0.16 | 0.13, 0.18 | .71 | ≤ .001 |
|  |  |  | substitute gratification | 0.01 | -0.02, 0.05 | .04 | .381 |
| Model b | .06 | ≤ .001 | ADHD | 0.11 | 0.09, 0.14 | .51 | ≤ .001 |
|  |  |  | substitute gratification | 0.01 | -0.02, 0.04 | .02 | .687 |
|  |  |  | SCL-90-R GSI | 0.06 | 0.04, 0.08 | .32 | ≤ .001 |
| Model c | .01 | .061 | ADHD* substitute gratification | 0.00 | 0.00, 0.00 | .49 | .061 |
| Model a | .00 | .897 | ADHD | 0.16 | 0.13, 0.18 | .71 | ≤ .001 |
|  |  |  | search for self-affirmation | 0.00 | -0.04, 0.03 | -.01 | .897 |
| Model b | .06 | ≤ .001 | ADHD | 0.11 | 0.08, 0.13 | .50 | ≤ .001 |
|  |  |  | search for self-affirmation | -0.01 | -0.04, 0.02 | -.03 | .593 |
|  |  |  | SCL-90-R GSI | 0.06 | 0.04, 0.08 | .33 | ≤ .001 |
| Model c | .00 | .164 | ADHD* search for self-affirmation | 0.00 | 0.00, 0.00 | .40 | .164 |
| Model a | .01 | .029 | ADHD | 0.15 | 0.12, 0.17 | .67 | ≤ .001 |
|  |  |  | situation control | -0.04 | -0.07, 0.00 | -.12 | .029 |
| Model b | .06 | ≤ .001 | ADHD | 0.11 | 0.08, 0.13 | .48 | ≤ .001 |
|  |  |  | situation control | -0.03 | -0.06, 0.00 | -.09 | .072 |
|  |  |  | SCL-90-R GSI | 0.06 | 0.04, 0.08 | .31 | ≤ .001 |
| Model c | .00 | .344 | ADHD*situation control | 0.00 | 0.00, 0.00 | .20 | .344 |
| Model a | .01 | .126 | ADHD | 0.15 | 0.13, 0.18 | .70 | ≤ .001 |
|  |  |  | reaction control | -0.03 | -0.07, 0.01 | -.08 | .126 |
| Model b | .06 | ≤ .001 | ADHD | 0.11 | 0.08, 0.14 | .50 | ≤ .001 |
|  |  |  | reaction control | -0.03 | -0.06, 0.01 | -.07 | .134 |
|  |  |  | SCL-90-R GSI | 0.06 | 0.04, 0.08 | .32 | ≤ .001 |
| Model c | .00 | .453 | ADHD* reaction control | 0.00 | 0.00, 0.00 | .18 | .453 |
| Model a | .01 | .012 | ADHD | 0.14 | 0.12, 0.17 | .65 | ≤ .001 |
|  |  |  | positive self-instructions | -0.04 | -0.08, -0.01 | -.13 | .012 |
| Model b | .05 | ≤ .001 | ADHD | 0.11 | 0.08, 0.13 | .48 | ≤ .001 |
|  |  |  | positive self-instructions | -0.03 | -0.05, 0.00 | -.09 | .066 |
|  |  |  | SCL-90-R GSI | 0.06 | 0.04, 0.08 | .31 | ≤ .001 |
| Model c | .00 | .499 | ADHD* positive self-instructions | 0.00 | 0.00, 0.00 | .14 | .499 |
| Model a | .00 | .187 | ADHD | 0.15 | 0.13, 0.18 | .70 | ≤ .001 |
|  |  |  | need for social support | -0.03 | -0.07, 0.01 | -.07 | .187 |
| Model b | .06 | ≤ .001 | ADHD | 0.11 | 0.08, 0.14 | .50 | ≤ .001 |
|  |  |  | need for social support | -0.03 | -0.07, 0.01 | -.08 | .107 |
|  |  |  | SCL-90-R GSI | 0.06 | 0.04, 0.08 | .33 | ≤ .001 |
| Model c | .00 | .881 | ADHD* need for social support | 0.00 | 0.00, 0.00 | .05 | .881 |
| Model a | .01 | .017 | ADHD | 0.15 | 0.12, 0.17 | .67 | ≤ .001 |
|  |  |  | avoidance | 0.04 | 0.01, 0.08 | .12 | .017 |
| Model b | .05 | ≤ .001 | ADHD | 0.11 | 0.08, 0.14 | .49 | ≤ .001 |
|  |  |  | avoidance | 0.02 | -0.01, 0.06 | .07 | .144 |
|  |  |  | SCL-90-R GSI | 0.06 | 0.03, 0.08 | .30 | ≤ .001 |
| Model c | .00 | .239 | ADHD*avoidance | 0.00 | 0.00, 0.00 | .32 | .239 |
| Model a | .03 | ≤ .001 | ADHD | 0.13 | 0.10, 0.15 | .58 | ≤ .001 |
|  |  |  | escape | 0.06 | 0.03, 0.08 | .21 | ≤ .001 |
| Model b | .04 | ≤ .001 | ADHD | 0.10 | 0.07, 0.13 | .45 | ≤ .001 |
|  |  |  | escape | 0.04 | 0.01, 0.07 | .14 | .021 |
|  |  |  | SCL-90-R GSI | 0.05 | 0.03, 0.08 | .28 | ≤ .001 |
| Model c | .00 | .904 | ADHD*escape | 0.00 | 0.00, 0.00 | .03 | .904 |
| Model a | .04 | ≤ .001 | ADHD | 0.12 | 0.09, 0.15 | .55 | ≤ .001 |
|  |  |  | social withdrawal | 0.07 | 0.04, 0.11 | .26 | ≤ .001 |
| Model b | .04 | ≤ .001 | ADHD | 0.09 | 0.06, 0.12 | .42 | ≤ .001 |
|  |  |  | social withdrawal | 0..05 | 0.02, 0.09 | .19 | .002 |
|  |  |  | SCL-90-R GSI | 0.05 | 0.03, 0.07 | .28 | ≤ .001 |
| Model c | .00 | .926 | ADHD*social withdrawal | 0.00 | 0.00, 0.00 | -.03 | .926 |
| Model a | .00 | .561 | ADHD | 0.15 | 0.13, 0.18 | .60 | ≤ .001 |
|  |  |  | rumination | 0.01 | -0.02, 0.04 | .03 | .561 |
| Model b | .06 | ≤ .001 | ADHD | 0.11 | 0.09, 0.14 | .52 | ≤ .001 |
|  |  |  | rumination | -0.01 | -0.05, 0.02 | -.05 | .389 |
|  |  |  | SCL-90-R GSI | 0.06 | 0.04, 0.09 | .34 | ≤ .001 |
| Model c | .00 | .801 | ADHD*rumination | 0.00 | 0.00, 0.00 | .07 | .801 |
| Model a | .03 | ≤ .001 | ADHD | 0.12 | 0.10, 0.15 | .56 | ≤ .001 |
|  |  |  | resignation | 0.06 | 0.03, 0.09 | .22 | ≤ .001 |
| Model b | .04 | ≤ .001 | ADHD | 0.10 | 0.07, 0.13 | .45 | ≤ .001 |
|  |  |  | resignation | 0.03 | 0.00, 0.07 | .12 | .059 |
|  |  |  | SCL-90-R GSI | 0.05 | 0.03, 0.08 | .28 | ≤ .001 |
| Model c | .00 | .605 | ADHD*resignation | 0.00 | 0.00, 0.00 | .16 | .605 |
| Model a | .02 | .002 | ADHD | 0.14 | 0.11, 0.16 | .62 | ≤ .001 |
|  |  |  | self-pity | 0.05 | 0.02, 0.09 | .13 | .002 |
| Model b | .04 | ≤ .001 | ADHD | 0.11 | 0.08, 0.13 | .49 | ≤ .001 |
|  |  |  | self-pity | 0.02 | -0.01, 0.06 | .07 | .202 |
|  |  |  | SCL-90-R GSI | 0.05 | 0.03, 0.08 | .29 | ≤ .001 |
| Model c | .00 | .253 | ADHD*self-pity | 0.00 | 0.00, 0.00 | .33 | .253 |
| Model a | .01 | .144 | ADHD | 0.15 | 0.12, 0.17 | .66 | ≤ .001 |
|  |  |  | self-blame | 0.02 | -0.01, 0.06 | .09 | .144 |
| Model b | .05 | ≤ .001 | ADHD | 0.11 | 0.08, 0.14 | .51 | ≤ .001 |
|  |  |  | self-blame | 0.00 | -0.03, 0.03 | .00 | .964 |
|  |  |  | SCL-90-R GSI | 0.06 | 0.04, 0.08 | .32 | ≤ .001 |
| Model c | .00 | .915 | ADHD*self-blame | 0.00 | 0.00, 0.00 | .03 | .915 |
| Model a | .02 | .002 | ADHD | 0.13 | 0.10, 0.16 | .58 | ≤ .001 |
|  |  |  | aggression | 0.06 | 0.02, 0.09 | .20 | .002 |
| Model b | .05 | ≤ .001 | ADHD | 0.09 | 0.07, 0.12 | .43 | ≤ .001 |
|  |  |  | aggression | 0.04 | 0.01, 0.08 | .15 | .016 |
|  |  |  | SCL-90-R GSI | 0.06 | 0.03, 0.08 | .30 | ≤ .001 |
| Model c | .00 | .897 | ADHD*aggression | 0.00 | 0.00, 0.00 | .04 | .897 |
| Model a | .00 | .330 | ADHD | 0.15 | 0.12, 0.17 | .68 | ≤ .001 |
|  |  |  | drug use | 0.02 | -0.02, 0.05 | .06 | .330 |
| Model b | .06 | ≤ .001 | ADHD | 0.11 | 0.08, 0.14 | .51 | ≤ .001 |
|  |  |  | drug use | 0.00 | -0.04, 0.03 | .00 | .942 |
|  |  |  | SCL-90-R GSI | 0.06 | 0.04, 0.08 | .32 | ≤ .001 |
| Model c | .00 | .963 | ADHD*drug use | 0.00 | 0.00, 0.00 | -.02 | .963 |
| *Note.* All models include age, sex, and IQ as control variables. Model c includes all mentioned variables, only the interaction term is displayed. | | | | | | | |
