# Supplementary material for: The role of stress coping strategies for life impairments in ADHD
Source: J Neural Transm (Vienna). 2021 Mar 9;128(7):981–92. doi: 10.1007/s00702-021-02311-5 (PMC8295144; doi:10.1007/s00702-021-02311-5)
Supplement: Supplementary file 2 — Supplementary file2 (DOCX 26 KB) [file 702_2021_2311_MOESM2_ESM.docx]

Online Supplement 2: Associations between ADHD, stress coping strategies, and Sheehan Disability Scale: social life impairments.

|  | Δ*R²* | *p* | Predictor | *B* | 95%*CI* | ß | *p* |
| --- | --- | --- | --- | --- | --- | --- | --- |
|  | .41 | ≤ .001 | ADHD | 0.14 | 0.12, 0.16 | .65 | ≤ .001 |
| Model a | .03 | ≤ .001 | ADHD | 0.13 | 0.11, 0.15 | .60 | ≤ .001 |
|  |  |  | minimization | -0.06 | -0.10, -0.03 | -.19 | ≤ .001 |
| Model b | .07 | ≤ .001 | ADHD | 0.08 | 0.06, 0.11 | .38 | ≤ .001 |
|  |  |  | minimization | -0.06 | -0.09, -0.03 | -.18 | ≤ .001 |
|  |  |  | SCL-90-R GSI | 0.07 | .0.04, 0.09 | .36 | ≤ .001 |
| Model c | .00 | .617 | ADHD*minimization | 0.00 | 0.00, 0.00 | .12 | .617 |
| Model a | .02 | .011 | ADHD | 0.13 | 0.11, 0.15 | .60 | ≤ .001 |
|  |  |  | self-aggrandizement by comparison with  others | -0.05 | -0.08, -0.01 | -.15 | .011 |
| Model b | .07 | ≤ .001 | ADHD | 0.09 | .0.06, 0.11 | .40 | ≤ .001 |
|  |  |  | self-aggrandizement by comparison with  others | -0.03 | -0.06, 0.00 | -.10 | .081 |
|  |  |  | SCL-90-R GSI | 0.06 | 0.04, 0.09 | .34 | ≤ .001 |
| Model c | .00 | .198 | ADHD* self-aggrandizement by comparison  with others | 0.00 | 0.00, 0.00 | -.29 | .198 |
| Model a | .00 | .615 | ADHD | 0.14 | 0.12, 0.16 | .65 | ≤ .001 |
|  |  |  | denial of guild | -0.01 | -0.03, 0.03 | -.03 | .615 |
| Model b | .08 | ≤ .001 | ADHD | 0.09 | 0.06, 0.12 | .42 | ≤ .001 |
|  |  |  | denial of guild | -0.01 | -0.03, 0.03 | -.02 | .688 |
|  |  |  | SCL-90-R GSI | 0.07 | 0.04, 0.09 | .37 | ≤ .001 |
| Model c | .00 | .321 | ADHD*denial of guild | 0.00 | 0.00, 0.00 | .23 | .321 |
| Model a | .01 | .033 | ADHD | 0.14 | 0.12, 0.16 | .65 | ≤ .001 |
|  |  |  | distraction | -0.04 | -0.08, 0.00 | -.12 | .033 |
| Model b | .08 | ≤ .001 | ADHD | 0.09 | 0.06, 0.12 | .41 | ≤ .001 |
|  |  |  | distraction | -0.05 | -0.08, -0.01 | -.13 | .009 |
|  |  |  | SCL-90-R GSI | 0.07 | 0.05, 0.09 | .38 | ≤ .001 |
| Model c | .00 | .776 | ADHD* distraction | 0.00 | 0.00, 0.00 | -.08 | .776 |
| Model a | .00 | .941 | ADHD | 0.14 | 0.12, 0.16 | .65 | ≤ .001 |
|  |  |  | substitute gratification | 0.00 | -0.03, 0.04 | .00 | .941 |
| Model b | .08 | ≤ .001 | ADHD | 0.09 | 0.06, 0.12 | .42 | ≤ .001 |
|  |  |  | substitute gratification | -0.01 | -0.03, 0.02 | -.03 | .625 |
|  |  |  | SCL-90-R GSI | 0.07 | 0.05, 0.09 | .37 | ≤ .001 |
| Model c | .01 | .032 | ADHD* substitute gratification | 0.00 | 0.00, 0.00 | .61 | .032 |
| Model a | .01 | .044 | ADHD | 0.14 | 0.11, 0.16 | .64 | ≤ .001 |
|  |  |  | search for self-affirmation | -0.04 | -0.08, 0.00 | -.11 | .044 |
| Model b | .08 | ≤ .001 | ADHD | 0.09 | 0.06, 0.11 | .40 | ≤ .001 |
|  |  |  | search for self-affirmation | -0.05 | -9,98, -0.01 | -.13 | .009 |
|  |  |  | SCL-90-R GSI | 0.07 | 0.05, 0.09 | .38 | ≤ .001 |
| Model c | .00 | .659 | ADHD* search for self-affirmation | 0.00 | 0.00, 0.00 | .13 | .659 |
| Model a | .02 | .014 | ADHD | 0.13 | 0.11, 0.15 | .60 | ≤ .001 |
|  |  |  | situation control | -0.04 | -0.08, -0.01 | -.14 | .014 |
| Model b | .07 | ≤ .001 | ADHD | 0.08 | 0.06, 0.11 | .39 | ≤ .001 |
|  |  |  | situation control | -0.04 | -0.07, 0.00 | -.11 | .039 |
|  |  |  | SCL-90-R GSI | 0.07 | 0.04, 0.09 | .36 | ≤ .001 |
| Model c | .00 | .319 | ADHD*situation control | 0.00 | 0.00, 0.00 | .23 | .319 |
| Model a | .03 | .002 | ADHD | 0.14 | 0.11, 0.16 | .63 | ≤ .001 |
|  |  |  | reaction control | -0.07 | -0.11, -0.03 | -.17 | .002 |
| Model b | .08 | ≤ .001 | ADHD | 0.09 | 0.06, 0.12 | .41 | ≤ .001 |
|  |  |  | reaction control | -0.07 | -0.10, -0.03 | -.16 | ≤ .001 |
|  |  |  | SCL-90-R GSI | 0.07 | 0.04, 0.09 | .37 | ≤ .001 |
| Model c | .00 | .490 | ADHD* reaction control | 0.00 | 0.00, 0.00 | .18 | .490 |
| Model a | .04 | ≤ .001 | ADHD | 0.12 | 0.10, 0.15 | .57 | ≤ .001 |
|  |  |  | positive self-instructions | -0.07 | -0.11, -0.03 | -.21 | ≤ .001 |
| Model b | .06 | ≤ .001 | ADHD | 0.08 | 0.05, 0.11 | .38 | ≤ .001 |
|  |  |  | positive self-instructions | -0.05 | -0.09, -0.02 | -.17 | .002 |
|  |  |  | SCL-90-R GSI | 0.06 | 0.04, 0.09 | .34 | ≤ .001 |
| Model c | .00 | .687 | ADHD* positive self-instructions | 0.00 | 0.00, 0.00 | -.09 | .687 |
| Model a | .00 | .421 | ADHD | 0.14 | 0.12, 0.16 | .65 | ≤ .001 |
|  |  |  | need for social support | -0.02 | -0.06, 0.03 | -.04 | .421 |
| Model b | .08 | ≤ .001 | ADHD | 0.09 | 0.06, 0.12 | .42 | ≤ .001 |
|  |  |  | need for social support | -0.02 | -0.06, 0.02 | -.05 | .277 |
|  |  |  | SCL-90-R GSI | 0.07 | 0.05, 0.09 | .37 | ≤ .001 |
| Model c | .00 | .719 | ADHD* need for social support | 0.00 | 0.00, 0.00 | .12 | .719 |
| Model a | .01 | .136 | ADHD | 0.14 | 0.11, 0.16 | .63 | ≤ .001 |
|  |  |  | avoidance | 0.03 | -0.01, 0.06 | .08 | .136 |
| Model b | .07 | ≤ .001 | ADHD | 0.09 | 0.06, 0.12 | .42 | ≤ .001 |
|  |  |  | avoidance | 0.01 | -0.03, 0.04 | .02 | .659 |
|  |  |  | SCL-90-R GSI | 0.07 | 0.04, 0.09 | .37 | ≤ .001 |
| Model c | .01 | .037 | ADHD*avoidance | 0.00 | 0.00, 0.00 | .60 | .037 |
| Model a | .05 | ≤ .001 | ADHD | 0.11 | 0.08, 0.13 | .49 | ≤ .001 |
|  |  |  | escape | 0.07 | 0.04, 0.10 | .27 | ≤ .001 |
| Model b | .05 | ≤ .001 | ADHD | 0.08 | 0.05, 0.10 | .35 | ≤ .001 |
|  |  |  | escape | 0.05 | 0.01, 0.08 | .13 | .005 |
|  |  |  | SCL-90-R GSI | 0.06 | 0.03, 0.08 | .32 | ≤ .001 |
| Model c | .01 | .067 | ADHD*escape | 0.00 | 0.00, 0.00 | .54 | .067 |
| Model a | .06 | ≤ .001 | ADHD | 0.10 | 0.07, 0.13 | .45 | ≤ .001 |
|  |  |  | social withdrawal | 0.09 | 0.05, 0.12 | .32 | ≤ .001 |
| Model b | .05 | ≤ .001 | ADHD | 0.07 | 0.04, 0.10 | .31 | ≤ .001 |
|  |  |  | social withdrawal | 0.07 | 0.03, 0.10 | .24 | ≤ .001 |
|  |  |  | SCL-90-R GSI | 0.06 | 0.03, 0.08 | .31 | ≤ .001 |
| Model c | .91 | .039 | ADHD*social withdrawal | 0.00 | 0.00, 0.00 | .66 | .039 |
| Model a | .02 | .021 | ADHD | 0.13 | 0.10, 0.15 | .59 | ≤ .001 |
|  |  |  | rumination | 0.04 | 0.01, 0.08 | .14 | .021 |
| Model b | .07 | ≤ .001 | ADHD | 0.09 | 0.06, 0.12 | .41 | ≤ .001 |
|  |  |  | rumination | 0.02 | -0.02., 0.05 | .06 | .339 |
|  |  |  | SCL-90-R GSI | 0.06 | 0.04, 0.09 | .35 | ≤ .001 |
| Model c | .01 | .021 | ADHD*rumination | 0.00 | 0.00, 0.01 | .73 | .021 |
| Model a | .05 | ≤ .001 | ADHD | 0.10 | 0.08, 0.13 | .46 | ≤ .001 |
|  |  |  | resignation | 0.08 | 0.04, 0.11 | .29 | ≤ .001 |
| Model b | .05 | ≤ .001 | ADHD | 0.07 | 0.04, 0.10 | .34 | ≤ .001 |
|  |  |  | resignation | 0.05 | 0.01, 0.09 | .19 | .007 |
|  |  |  | SCL-90-R GSI | 0.06 | 0.03, 0.08 | .31 | ≤ .001 |
| Model c | .01 | .014 | ADHD*resignation | 0.00 | 0.00, 0.01 | .81 | .014 |
| Model a | .02 | .020 | ADHD | 0.13 | 0.10, 0.15 | .58 | ≤ .001 |
|  |  |  | self-pity | 0.04 | 0.01, 0.08 | .13 | .020 |
| Model b | .06 | ≤ .001 | ADHD | 0.09 | 0.06, 0.12 | .42 | ≤ .001 |
|  |  |  | self-pity | 0.01 | -0.03, 0.04 | .02 | .768 |
|  |  |  | SCL-90-R GSI | 0.07 | 0.04, 0.09 | .36 | ≤ .001 |
| Model c | .01 | .151 | ADHD*self-pity | 0.00 | 0.00, 0.00 | .44 | .151 |
| Model a | .01 | .029 | ADHD | 0.13 | 0.10, 0.15 | .58 | ≤ .001 |
|  |  |  | self-blame | 0.03 | 0.00, 0.07 | .13 | .029 |
| Model b | .07 | ≤ .001 | ADHD | 0.09 | 0.06, 0.12 | .41 | ≤ .001 |
|  |  |  | self-blame | 0.01 | -0.02, 0.04 | .04 | .510 |
|  |  |  | SCL-90-R GSI | 0.07 | 0.04, 0.09 | .36 | ≤ .001 |
| Model c | .00 | .220 | ADHD*self-blame | 0.00 | 0.00, 0.00 | .39 | .220 |
| Model a | .01 | .032 | ADHD | 0.12 | 0.09, 0.15 | .60 | ≤ .001 |
|  |  |  | aggression | 0.04 | 0.00, 0.08 | .15 | .032 |
| Model b | .07 | ≤ .001 | ADHD | 0.08 | 0.05, 0.11 | .38 | ≤ .001 |
|  |  |  | aggression | 0.02 | -0.01, 0.06 | .09 | .190 |
|  |  |  | SCL-90-R GSI | 0.07 | 0.05, 0.09 | .36 | ≤ .001 |
| Model c | .00 | .588 | ADHD*aggression | 0.00 | 0.00, 0.00 | .17 | .588 |
| Model a | .00 | .448 | ADHD | 0.14 | 0.11, 0.16 | .63 | ≤ .001 |
|  |  |  | drug use | 0.02 | -0.02, 0.05 | .05 | .448 |
| Model b | .08 | ≤ .001 | ADHD | 0.09 | 0.06, 0.12 | .43 | ≤ .001 |
|  |  |  | drug use | -0.01 | -0.04, 0.03 | -.02 | .707 |
|  |  |  | SCL-90-R GSI | 0.07 | 0.05, 0.09 | .38 | ≤ .001 |
| Model c | .00 | .492 | ADHD*drug use | 0.00 | 0.00, 0.00 | .24 | .492 |
| *Note.* All models include age, sex, and IQ as control variables. Model c includes all mentioned variables, only the interaction term is displayed. | | | | | | | |
