# Supplementary material for: The role of stress coping strategies for life impairments in ADHD
Source: J Neural Transm (Vienna). 2021 Mar 9;128(7):981–92. doi: 10.1007/s00702-021-02311-5 (PMC8295144; doi:10.1007/s00702-021-02311-5)
Supplement: Supplementary file 3 — Supplementary file3 (DOCX 26 KB) [file 702_2021_2311_MOESM3_ESM.docx]

Online Supplement 1: Associations between ADHD, stress coping strategies, and Sheehan Disability Scale: work/school impairments.

|  | Δ*R²* | *p* | Predictor | *B* | 95%*CI* | ß | *p* |
| --- | --- | --- | --- | --- | --- | --- | --- |
|  | .44 | ≤ .001 | ADHD | 0.16 | 0.13, 0.18 | .66 | ≤ .001 |
| Model a | .00 | .205 | ADHD | 0.15 | 0.13, 0.18 | .65 | ≤ .001 |
|  |  |  | minimization | -0.03 | -0.06, 0.01 | -.07 | .205 |
| Model b | .03 | ≤ .001 | ADHD | 0.12 | 0.09, 0.15 | .51 | ≤ .001 |
|  |  |  | minimization | -0.02 | -0.06, 0.02 | -.06 | .257 |
|  |  |  | SCL-90-R GSI | 0.05 | 0.02, 0.07 | .23 | ≤ .001 |
| Model c | .00 | .767 | ADHD*minimization | 0.00 | 0.00, 0.00 | .08 | .767 |
| Model a | .01 | .110 | ADHD | 0.15 | 0.12, 0.17 | .63 | ≤ .001 |
|  |  |  | self-aggrandizement by comparison with  others | -0.03 | -0.07, 0.01 | -.02 | .783 |
| Model b | .03 | ≤ .001 | ADHD | 0.12 | 0.09, 0.15 | .51 | ≤ .001 |
|  |  |  | self-aggrandizement by comparison with  others | -0.02 | -0.06, 0.02 | -.06 | .200 |
|  |  |  | SCL-90-R GSI | 0.04 | 0.02, 0.07 | .22 | ≤ .001 |
| Model c | .00 | .691 | ADHD* self-aggrandizement by comparison with others | 0.00 | 0.00, 0.00 | -.09 | .691 |
| Model a | .00 | .232 | ADHD | 0.15 | 0.13, 0.18 | .66 | ≤ .001 |
|  |  |  | denial of guild | -0.02 | -0.06, 0.01 | -.06 | .232 |
| Model b | .03 | ≤ .001 | ADHD | 0.12 | 0.09, 0.15 | .52 | ≤ .001 |
|  |  |  | denial of guild | -0.02 | -0.05, 0.01 | -.06 | .253 |
|  |  |  | SCL-90-R GSI | 0.05 | 0.02, 0.07 | .23 | ≤ .001 |
| Model c | .00 | .553 | ADHD*denial of guild | 0.00 | 0.00, 0.00 | -.14 | .553 |
| Model a | .01 | .063 | ADHD | 0.15 | 0.13, 0.18 | .66 | ≤ .001 |
|  |  |  | distraction | -0.04 | -0.07, 0.00 | -.10 | .063 |
| Model b | .03 | ≤ .001 | ADHD | 0.12 | 0.09, 0.15 | .51 | ≤ .001 |
|  |  |  | distraction | -0.04 | -0.08, 0.00 | -.11 | .036 |
|  |  |  | SCL-90-R GSI | 0.05 | 0.02, 0.07 | .24 | ≤ .001 |
| Model c | .00 | .380 | ADHD* distraction | 0.00 | 0.00, 0.00 | -.23 | .380 |
| Model a | .00 | .348 | ADHD | 0.16 | 0.13, 0.18 | .67 | ≤ .001 |
|  |  |  | substitute gratification | 0.02 | -0.02, 0.05 | .05 | .348 |
| Model b | .03 | ≤ .001 | ADHD | 0.12 | 0.09, 0.15 | .53 | ≤ .001 |
|  |  |  | substitute gratification | 0.01 | -0.02, 0.05 | .03 | .534 |
|  |  |  | SCL-90-R GSI | 0.05 | 0.02, 0.05 | .22 | ≤ .001 |
| Model c | .00 | .226 | ADHD* substitute gratification | 0.00 | 0.00, 0.00 | .35 | .226 |
| Model a | .00 | .676 | ADHD | 0.16 | 0.13, 0.18 | .66 | ≤ .001 |
|  |  |  | search for self-affirmation | -0.01 | -0.05, 0.03 | -.02 | .676 |
| Model b | .03 | ≤ .001 | ADHD | 0.12 | 0.09, 0.15 | .51 | ≤ .001 |
|  |  |  | search for self-affirmation | -0.01 | -0.05, 0.03 | -.04 | .493 |
|  |  |  | SCL-90-R GSI | 0.05 | 0.02, 0.07 | .23 | ≤ .001 |
| Model c | .00 | .517 | ADHD* search for self-affirmation | 0.00 | 0.00, 0.00 | .20 | .517 |
| Model a | .01 | .115 | ADHD | 0.15 | 0.12, 0.17 | .63 | ≤ .001 |
|  |  |  | situation control | -0.03 | -0.07, 0.01 | -.09 | .115 |
| Model b | .03 | ≤ .001 | ADHD | 0.12 | 0.09, 0.15 | .50 | ≤ .001 |
|  |  |  | situation control | -0.02 | -0.06, 0.01 | -.07 | .200 |
|  |  |  | SCL-90-R GSI | 0.04 | 0.02, 0.07 | .22 | ≤ .001 |
| Model c | .01 | .027 | ADHD*situation control | 0.00 | 0.00, 0.01 | .51 | .027 |
| Model a | .00 | .206 | ADHD | 0.15 | 0.13, 0.18 | .66 | ≤ .001 |
|  |  |  | reaction control | -0.03 | -0.07, 0.02 | -.07 | .206 |
| Model b | .03 | ≤ .001 | ADHD | 0.12 | 0.09, 0.15 | .52 | ≤ .001 |
|  |  |  | reaction control | -0.03 | -0.07, 0.02 | -.06 | .225 |
|  |  |  | SCL-90-R GSI | 0.05 | 0.02, 0.04 | .23 | ≤ .001 |
| Model c | .00 | .399 | ADHD* reaction control | 0.00 | 0.00, 0.00 | .22 | .399 |
| Model a | .02 | .010 | ADHD | 0.14 | 0.12, 0.17 | .61 | ≤ .001 |
|  |  |  | positive self-instructions | -0.05 | -0.09, -0.01 | -.14 | .010 |
| Model b | .02 | .002 | ADHD | 0.11 | 0.08, 0.15 | .49 | ≤ .001 |
|  |  |  | positive self-instructions | -0.04 | -0.08, 0.00 | -.12 | .035 |
|  |  |  | SCL-90-R GSI | 0.04 | 0.02, 0.07 | .21 | .002 |
| Model c | .00 | .837 | ADHD* positive self-instructions | 0.00 | 0.00, 0.00 | .05 | .837 |
| Model a | .00 | .333 | ADHD | 0.16 | 0.13, 0.18 | .67 | ≤ .001 |
|  |  |  | need for social support | 0.02 | -0.02, 0.07 | .05 | .333 |
| Model b | .03 | ≤ .001 | ADHD | 0.12 | 0.09, 0.15 | .53 | ≤ .001 |
|  |  |  | need for social support | 0.02 | -0.03, 0.06 | .04 | .390 |
|  |  |  | SCL-90-R GSI | 0.05 | 0.02, 0.07 | .23 | ≤ .001 |
| Model c | .00 | .983 | ADHD* need for social support | 0.00 | 0.00, 0.00 | .01 | .983 |
| Model a | .01 | .103 | ADHD | 0.15 | 0.12, 0.17 | .63 | ≤ .001 |
|  |  |  | avoidance | 0.03 | -0.01, 0.07 | .09 | .103 |
| Model b | .03 | .002 | ADHD | 0.12 | 0.09, 0.15 | .51 | ≤ .001 |
|  |  |  | avoidance | 0.02 | -0.02, 0.06 | .05 | .325 |
|  |  |  | SCL-90-R GSI | 0.04 | 0.02, 0.07 | .22 | .002 |
| Model c | .00 | .642 | ADHD*avoidance | 0.00 | 0.00, 0.00 | .14 | .642 |
| Model a | .04 | ≤ .001 | ADHD | 0.12 | 0.09, 0.15 | .51 | ≤ .001 |
|  |  |  | escape | 0.07 | 0.04, 0.10 | .25 | ≤ .001 |
| Model b | .01 | .015 | ADHD | 0.10 | 0.07, 0.13 | .44 | ≤ .001 |
|  |  |  | escape | 0.06 | 0.02, 0.09 | .20 | .002 |
|  |  |  | SCL-90-R GSI | 0.03 | 0.01, 0.06 | .16 | .015 |
| Model c | .00 | .372 | ADHD*escape | 0.00 | 0.00, 0.00 | -.27 | .372 |
| Model a | .02 | .007 | ADHD | 0.13 | 0.10, 0.16 | .55 | ≤ .001 |
|  |  |  | social withdrawal | 0.05 | 0.02, 0.09 | .18 | .007 |
| Model b | .02 | .004 | ADHD | 0.11 | 0.07, 0.14 | .46 | ≤ .001 |
|  |  |  | social withdrawal | 0.04 | 0.00, 0.08 | .13 | .049 |
|  |  |  | SCL-90-R GSI | 0.04 | 0.01, 0.07 | .20 | .004 |
| Model c | .01 | .071 | ADHD*social withdrawal | 0.00 | -0.01, 0.00 | -.60 | .071 |
| Model a | .01 | .043 | ADHD | 0.14 | 0.12, 0.17 | .61 | ≤ .001 |
|  |  |  | rumination | 0.04 | 0.00, 0.07 | .12 | .043 |
| Model b | .02 | .003 | ADHD | 0.12 | 0.09, 0.15 | .50 | ≤ .001 |
|  |  |  | rumination | .02 | -0.02, 0.06 | .07 | .239 |
|  |  |  | SCL-90-R GSI | 0.04 | 0.01, 0.07 | .21 | .003 |
| Model c | .00 | .871 | ADHD*rumination | 0.00 | 0.00, 0.00 | .05 | .871 |
| Model a | .04 | ≤ .001 | ADHD | 0.12 | 0.09, 0.15 | .50 | ≤ .001 |
|  |  |  | resignation | 0.07 | 0.03, 0.11 | .25 | ≤ .001 |
| Model b | .01 | .018 | ADHD | 0.10 | 0.07, 0.13 | .43 | ≤ .001 |
|  |  |  | resignation | 0.06 | 0.02, 0.10 | .20 | .005 |
|  |  |  | SCL-90-R GSI | 0.03 | 0.01, 0.06 | .16 | .018 |
| Model c | .01 | .139 | ADHD*resignation | 0.00 | 0.00, 0.00 | -.50 | .139 |
| Model a | .01 | .047 | ADHD | 0.14 | 0.11, 0.17 | .60 | ≤ .001 |
|  |  |  | self-pity | 0.04 | 0.00, 0.08 | .12 | .047 |
| Model b | .02 | .004 | ADHD | 0.12 | 0.09, 0.15 | .51 | ≤ .001 |
|  |  |  | self-pity | 0.02 | -0.03, 0.06 | .05 | .452 |
|  |  |  | SCL-90-R GSI | 0.04 | 0.01, 0.07 | .21 | .004 |
| Model c | .01 | .060 | ADHD*self-pity | 0.00 | -0.01, 0.00 | -.59 | .060 |
| Model a | .01 | .161 | ADHD | 0.14 | 0.12, 0.17 | .62 | ≤ .001 |
|  |  |  | self-blame | 0.03 | -0.01, 0.06 | .09 | .161 |
| Model b | .03 | .002 | ADHD | 0.12 | 0.09, 0.15 | .51 | ≤ .001 |
|  |  |  | self-blame | 0.01 | -0.03, 0.04 | .03 | .673 |
|  |  |  | SCL-90-R GSI | 0.04 | 0.02, 0.07 | .22 | .002 |
| Model c | .00 | .593 | ADHD*self-blame | 0.00 | 0.00, 0.00 | -.17 | .593 |
| Model a | .01 | .03 | ADHD | 0.14 | 0.10, 0.17 | .57 | ≤ .001 |
|  |  |  | aggression | 0.04 | 0.00, 0.08 | .14 | .037 |
| Model b | .02 | .002 | ADHD | 0.11 | 0.08, 0.14 | .47 | ≤ .001 |
|  |  |  | aggression | 0.03 | -0.01, 0.07 | .10 | .122 |
|  |  |  | SCL-90-R GSI | 0.03 | 0.02, 0.07 | .21 | .002 |
| Model c | .01 | .151 | ADHD*aggression | 0.00 | 0.00, 0.00 | -.46 | .151 |
| Model a | .00 | .844 | ADHD | 0.15 | 0.13, 0.18 | .66 | ≤ .001 |
|  |  |  | drug use | 0.00 | -0.04, 0.05 | .01 | .844 |
| Model b | .03 | ≤ .001. | ADHD | 0.13 | 0.09, 0.16 | .53 | ≤ .001 |
|  |  |  | drug use | -0.01 | -0.05, 0.03 | -.03 | .600 |
|  |  |  | SCL-90-R GSI | 0.05 | 0.02, 0.07 | .24 | ≤ .001 |
| Model c | .01 | .166 | ADHD*drug use | 0.00 | 0.00, 0.00 | -.49 | .166 |
| *Note.* All models include age, sex, and IQ as control variables. Model c includes all mentioned variables, only the interaction term is displayed. | | | | | | | |
